# Enhancing Immunotherapeutic Response in Colorectal Cancer with a Neuropilin 1–Targeting Tumor-Penetrating Peptide

**DOI:** 10.1158/2767-9764.CRC-25-0619

**Published:** 2026-06-10

**Authors:** Atsushi Fukugaki, Shigeo Hisamori, Dean Thumkeo, Hotaka Kawai, Yugo Matsui, Yuki Teramoto, Yoshihisa Okuchi, Atsuhito Omori, Norio Miyamura, Yukihito Kuroda, Kazuki N. Sugahara, Kazutaka Obama

**Affiliations:** 1 Department of Surgery, Graduate School of Medicine, Kyoto University, Kyoto, Japan.; 2 Department of Immunopharmacology, Graduate School of Medicine, Kyoto University, Kyoto, Japan.; 3 Department of Oral Pathology and Medicine, Graduate School of Medicine, Dentistry and Pharmaceutical Sciences, Okayama University, Okayama, Japan.; 4 Department of Diagnostic Pathology, Graduate School of Medicine, Kyoto University, Kyoto, Japan.; 5 Department of Gastroenterological Surgery and Oncology, Medical Research Institute Kitano Hospital, Osaka, Japan.; 6 Department of Surgery, Columbia University Vagelos College of Physicians and Surgeons, New York, New York.

## Abstract

Immune checkpoint inhibitors (ICI) provide durable responses in a subset of colorectal cancers, yet benefit is largely confined to microsatellite instability–high (MSI-H) tumors. We investigated whether the tumor-penetrating peptide iRGD enhances anti–programmed cell death protein 1 (PD-1) treatment efficacy and examined the clinical relevance of neuropilin 1 (NRP1) expression. Syngeneic mouse models (MSI-H MC38 and microsatellite-stable CT26) were treated with an anti–PD-1 antibody and iRGD. Tumor cell and stromal NRP1 contributions were compared using NRP1-knockout colorectal cancer cell lines. NRP1 expression was analyzed using immunohistochemistry in a consecutive cohort of 110 patients with stage III colorectal cancer who underwent curative surgery. iRGD and anti–PD-1 antibody combination therapy enhanced antitumor effects in both colorectal cancer models, accompanied by increased intratumoral CD8^+^ T-cell abundance and improvements in their function. This effect persisted in tumors lacking tumor cell NRP1, suggesting that stromal NRP1 played a key role in mediating iRGD activity. In pathologic specimens from patients with colorectal cancer, widespread NRP1 expression was noted across tumor cells, the stromal compartment, and the vasculature, suggesting potential applicability of iRGD-based therapy in human colorectal cancer. Notably, stromal NRP1 expression was associated with poor overall and disease-free survival. Collectively, these findings position stromal NRP1 as a prognostic marker and an actionable entry point for iRGD-based ICI strategies, providing a strong rationale for their clinical development to extend the benefits of ICIs to broader colorectal cancer populations.

**Significance:**

We demonstrated that iRGD enhanced anti–PD-1 therapy efficacy in colorectal cancer models, improving intratumoral permeability and antitumor immune responses. NRP1 is broadly expressed across tumor compartments, and stromal, particularly endothelial, NRP1 is a key mediator of iRGD activity, associated with poor prognosis, representing a potential therapeutic entry point.

## Introduction

Colorectal cancer is a leading cause of cancer-related death worldwide (1). Although clinical outcomes have improved with significant advances in systemic therapies, including cytotoxic agents, molecular targeted therapies, and immune checkpoint inhibitors (ICI), the prognosis remains poor for patients with unresectable or metastatic disease. One of the key challenges in the treatment of solid tumors, including colorectal cancer, is limited tumor specificity and poor intratumoral penetration of therapeutics (2). These factors hinder systemic treatment from reaching its full potential and contribute to therapeutic resistance.

The tumor-penetrating peptide iRGD (internalizing RGD), which binds to αv integrins and neuropilin 1 (NRP1), has emerged as a promising strategy to enhance intratumoral delivery of coadministered therapeutics (3). iRGD promotes the selective accumulation and deep penetration of drugs into tumor tissue through a multistep process involving integrin binding, proteolytic cleavage exposing a CendR motif, and subsequent NRP1-mediated transcytosis (4–6). Its utility has been extensively demonstrated in various preclinical models, particularly in combination with cytotoxic chemotherapy agents (7–9).

In recent years, ICIs, particularly anti–programmed cell death protein 1 (anti–PD-1) and anti–programmed cell death ligand 1 (anti–PD-L1) antibodies, have emerged as groundbreaking therapies capable of achieving long-term survival in select cancer types. In colorectal cancer, however, the clinical benefit of ICIs remains largely confined to tumors with high microsatellite instability (MSI-H), which account for only a small subset of patients (10–12). Despite emerging clinical studies suggesting that under specific conditions, microsatellite-stable (MSS) colorectal cancers may also respond to ICIs (13, 14), the overall ICI efficacy in this population remains limited, highlighting the need for combination strategies that can enhance therapeutic responses.

The antitumor activity of ICIs is expected, at least in part, to depend on adequate availability within the tumor microenvironment, where immune modulation takes place (15). In solid tumors such as colorectal cancer, features of the tumor microenvironment that impair effective immune regulation, such as abnormal vasculature and dense stromal architecture, may also limit the local distribution of therapeutic antibodies and thereby compromise treatment efficacy. From this perspective, iRGD may provide a potential method to improve the intratumoral distribution of antibody-based immunotherapies. Separately, a recent study in gastric cancer reported that iRGD treatment facilitated lymphocyte infiltration and enhanced the antitumor activity of PD-1–deficient T cells (16), indicating that iRGD can also increase immune cell access to tumors in certain contexts. Nevertheless, whether iRGD can enhance the therapeutic efficacy of ICIs in colorectal cancer remains unknown.

The iRGD tumor penetration pathway is mediated by NRP1, which is associated with tumor progression and poor prognosis in several cancer types (17–19). However, the expression profile of NRP1 and its prognostic relevance in colorectal cancer remain controversial, likely due to methodologic heterogeneity and variations in patient cohorts (20–22). In addition, given that NRP1 is expressed in multiple cellular compartments within the tumor microenvironment (23, 24), including endothelial cells, fibroblasts, and immune cells, it is plausible that its functional role extends beyond tumor cell–intrinsic mechanisms.

In this study, we investigated whether the concurrent administration of iRGD augments the therapeutic efficacy of an anti–PD-1 antibody in syngeneic mouse models of colorectal cancer. To elucidate the functional contribution of NRP1, we used NRP1-knockout (KO) colorectal cancer cell lines to assess drug delivery and treatment responses *in vivo*. Furthermore, we evaluated the expression profile of NRP1 in epithelial and stromal compartments using specimens from a homogeneous cohort of patients with stage III colorectal cancer who underwent curative surgery. Collectively, these approaches provide mechanistic insights into the role of NRP1 in regulating therapeutic permeability and establish a rationale for developing iRGD-based combination strategies for colorectal cancer treatment.

## Materials and Methods

### Cell lines and reagents

MC38 (RRID: CVCL_B288) and CT26 (RRID: CVCL_7254) murine colon cancer cell lines were kindly provided by Dr. Yoshiro Itatani (Kyoto University). Cells were used at low passage numbers after thawing. Cell line authentication was not performed in this study, and all cells were routinely tested for *Mycoplasma* contamination using the LookOut Mycoplasma PCR Detection Kit (Sigma-Aldrich) and confirmed to be negative.

MC38 cells were cultured in Dulbecco’s Modified Eagle Medium (Nacalai Tesque, 08459-65), and CT26 cells were cultured in RPMI 1640 medium (Nacalai Tesque, 30264-85), both supplemented with 10% fetal bovine serum (MP Biomedicals, 2917354H) and 1% penicillin/streptomycin (FUJIFILM Wako, 168-23191) at 37°C in a humidified atmosphere containing 5% CO_2_. NRP1-KO MC38 (MC38-NRP1-KO) cells were generated using the CRISPR-Cas9 system with a px458 plasmid (RRID: Addgene_48138) encoding a guide RNA targeting the *NRP1* gene. Green fluorescent protein–positive cells were sorted by fluorescence-activated cell sorting, and single-cell clones were expanded and validated by Western blotting to confirm NRP1 deletion. As a control, MC38 cells were transfected with a nontargeting empty vector (MC38-EV). The iRGD peptide (acetyl-CRGDKGPDC-NH_2_; LifeTein, LT216216) and anti–PD-1 antibody (Bio X Cell, BE0146, RRID: AB_10949053) were used in this study.

### Immunoblotting

NRP1 protein levels were assessed via immunoblotting using lysates from MC38-NRP1-KO and MC38-EV cells. Cells were lysed in sodium dodecyl sulfate (SDS) sample buffer (1 mol/L Tris-HCl pH 6.8, 10% SDS, and 50% glycerol) containing protease and phosphatase inhibitors (Nacalai Tesque, 04080-11 and 07574-61). Lysates were separated by SDS-PAGE and transferred onto Immobilon-P membranes (Millipore). The membranes were blocked with Blocking One (Nacalai Tesque 03953-95) for 20 minutes at room temperature, followed by overnight incubation with an anti-NRP1 primary antibody (1:1,000, Abcam, ab81321, RRID: AB_1640739) at 4°C and a horseradish peroxidase–conjugated anti–rabbit IgG secondary antibody (1:500, Agilent, P0448, RRID: AB_2617138) for 30 minutes at room temperature. β-Actin was used as a loading control. Signals were detected using Pierce ECL Plus Western Blotting Substrate (Thermo Fisher Scientific, 32132) and visualized using an LAS-4000 system (FUJIFILM).

### Tumor treatment experiments

Animal experiments were approved by the Animal Research Committee of the Graduate School of Medicine, Kyoto University (approval number: Med Kyoto 24328), and were conducted in accordance with the ARRIVE guidelines. Six-week-old female C57BL/6 (RRID: MGI:2159769) and BALB/c mice (RRID: MGI:2161072) were purchased from Japan SLC, Inc. and CLEA Japan, Inc., respectively.

Mice were subcutaneously inoculated in the right flank with 1 × 10^6^ MC38 cells (C57BL/6) or 5 × 10^5^ CT26 cells (BALB/c) under isoflurane anesthesia. On days 6 and 7 after inoculation, the mice were randomly assigned to 4 treatment groups. Treatments included intraperitoneal injections of phosphate-buffered saline (PBS) or anti–PD-1 antibody (50 μg/mouse for MC38 and 100 μg/mouse for CT26) with or without intravenous iRGD (25 μmol/kg) treatment twice weekly for 3 to 4 weeks. Tumor volume was calculated as (length × width^2^)/2 and recorded twice a week.

In a separate study, C57BL/6 mice were subcutaneously inoculated with 2 × 10^6^ MC38-NRP1-KO cells in the right flank and treated with anti–PD-1 antibody (50 μg, intraperitoneally, weekly) and iRGD (25 μmol/kg, intravenously, twice weekly) starting on day 28. Tumor volumes were measured until day 52 when the mice were euthanized.

### *In vivo* permeability assay

Tumor permeability assays were performed using Evans blue dye according to a previously published protocol (5). We performed the tumor permeability assay in MC38 or CT26 tumor-bearing mice, as well as in mice bearing MC38-NRP1-KO or MC38-EV tumors. Tumor-bearing mice were anesthetized and then intravenously injected with 100 μL of 10 mg/mL Evans blue dye (MP Biomedicals, 151108), followed by iRGD (12.5 μmol/kg) or PBS alone after 5 minutes later. After 30 minutes, the mice were perfused with PBS and tissues were harvested. Evans blue was extracted from tissues using N,N-dimethylformamide (FUJIFILM Wako, 045-2916) at 37°C for 24 to 48 hours. Absorbance was measured at 600 nm using a spectrophotometer. Absorbance values were corrected by subtracting baseline values obtained from mice not injected with Evans blue and normalized to tissue weight (per 100 mg).

### CD8 immunohistochemistry in murine tumor models

To assess the spatial distribution of CD8^+^ T cells, formalin-fixed, paraffin-embedded (FFPE) sections were prepared from MC38 and CT26 tumors treated with PBS, iRGD, anti–PD-1 antibody, or their combination. Sections were stained with anti-CD8 antibody (1:200, Cell Signaling Technology, #98941, RRID: AB_2756376) following antigen retrieval using Tris–ethylenediaminetetraacetic acid (EDTA) buffer (pH 9.0) at 120°C for 8 minutes. Primary antibody incubation was performed overnight at 4°C, and staining was visualized using 3,3′-diaminobenzidine. For quantitative analysis, multiple tumors per group were analyzed, and 10 randomly selected fields per tumor were evaluated in a blinded manner.

To evaluate the activation status of CD8^+^ T cells, granzyme B immunohistochemistry (IHC) was performed in MC38 and CT26 tumors from mice treated with anti–PD-1 antibody alone or in combination with iRGD. Sections were stained with anti–granzyme B antibody (1:200, Abcam, ab255598, RRID: AB_2860567) following antigen retrieval using Tris-EDTA buffer (pH 9.0) at 95°C for 20 minutes. Subsequent IHC procedures were performed as described for CD8 IHC.

### Flow cytometry

MC38 and CT26 tumors treated with PBS, iRGD, anti–PD-1 antibody, or their combination were dissociated into single-cell suspensions. Cells were stained with a fixable viability dye (Invitrogen, L34955) and blocked with an Fc blocking solution (BD Biosciences, 553141, RRID: AB_394656), followed by surface staining with antibodies against CD45 (Thermo Fisher Scientific, 11-0451-82, RRID: AB_465050), CD4 (BioLegend, 100540, RRID: AB_893326), CD8a (BioLegend, 100712, RRID: AB_312751), and CD25 (Thermo Fisher Scientific, 12-0251-82, RRID: AB_465607). Intracellular staining for Foxp3 (Thermo Fisher Scientific, 17-5773-82, RRID: AB_469457) was performed using a standard permeabilization kit (eBioscience, 00-5523-00). Data were acquired using a flow cytometer and analyzed using FlowJo software (BD Biosciences, RRID: SCR_008520).

### Immunofluorescence

Paraffin-embedded tumor sections from MC38- and CT26-bearing mice treated with anti–PD-1 antibody alone or anti–PD-1 antibody plus iRGD were stained to assess T-cell infiltration and proliferative status. Sections were incubated with anti-CD8 antibody (1:2,000, Abcam, ab217344, RRID: AB_2890649) and anti–Ki-67 antibody (1:400, Cell Signaling Technology, #12202, RRID: AB_2620142). Signal amplification was performed using the Tyramide Signal Amplification Plus system (PerkinElmer, NEL754001KT), with CD8 and Ki-67 signals visualized using Cyanine5 and fluorescein fluorophores, respectively. Nuclei were counterstained with 4′,6-diamidino-2-phenylindole (DAPI).

Paraffin-embedded sections from MC38-NRP1-KO and MC38-EV tumors were stained to evaluate NRP1 expression and tumor vasculature. Sections were incubated with anti-NRP1 antibody (1:100, R&D Systems, AF566, RRID: AB_355445) and anti-CD31 antibody (1:100, Cell Signaling Technology, #77699, RRID: AB_2722705), followed by Alexa Fluor 488– and Alexa Fluor 594–conjugated secondary antibodies (Thermo Fisher Scientific, A-11055, RRID: AB_2534102 and A-11012, RRID: AB_2534079).

All images were acquired using a BZ-X800 fluorescence microscope (Keyence, RRID: SCR_023617).

### Patients

We retrospectively analyzed 110 patients with stage III colorectal cancer who underwent primary tumor resection at Kyoto University Hospital between April 2014 and March 2018. Their colorectal cancer diagnosis and staging were confirmed by pathologic examination according to the eighth edition of the Union for International Cancer Control Tumor–Node–Metastasis Classification. The study protocol was approved by the Ethics Committee of the Graduate School and Faculty of Medicine, Kyoto University (approval number: R2351-4), and written informed consent was obtained from all patients, with an opt-out option provided. This study was conducted in accordance with the Declaration of Helsinki.

### NRP1 IHC in human colorectal cancer tissues

FFPE tumor sections were stained with the VENTANA BenchMark ULTRA system (Roche Diagnostics, RRID: SCR_013652) using anti-NRP1 antibody (1:100, Abcam, ab81321, RRID: AB_1640739). Signals were detected using a polymer-based system. Tumor cell staining intensity was scored on a scale of 0 to 3 and multiplied by the percentage of stained cells to obtain a composite score. High expression was defined at a threshold of 65, determined from the distribution of expression scores. Stromal staining was assessed with moderate or strong staining classified as high expression based on the predominant intensity, as stromal NRP1 expression was relatively homogeneous and involved multiple cell types that rendered quantitative scoring impractical. Each slide was independently assessed by a pathologist and 2 surgeons, all of whom were blinded to the clinical data.

To further characterize stromal NRP1 expression at the cellular level, serial sections from selected cases were stained for NRP1, CD31, and α-SMA. NRP1 staining was performed using the same conditions. CD31 and α-SMA staining was performed using anti-CD31 antibody (1:30, Leica, NCL-CD31-1A10, RRID: AB_442060) and anti–α-SMA antibody (1:1,000, Sigma-Aldrich, A2547, RRID: AB_476701), respectively. CD31-positive vascular structures were used to define vascular-associated areas, whereas nonvascular α-SMA–positive stromal areas were evaluated as cancer-associated fibroblast (CAF)–rich areas.

### Statistical analysis

Data are presented as mean ± SEM. GraphPad Prism (version 10; GraphPad Software, RRID: SCR_002798) and JMP (version 18; SAS Institute, RRID: SCR_014242) were used for data analysis. Group differences were analyzed using the Student *t* test or Mann–Whitney U test. For comparisons between more than 2 groups, either one-way analysis of variance (ANOVA) followed by the Tukey *post hoc* test or the Kruskal–Wallis test followed by the Dunn multiple comparisons test was used, depending on the data distribution. Tumor volume over time was analyzed using a two-way ANOVA. Kaplan–Meier survival curves were compared using the log-rank test. Cox proportional hazards models were used for univariate and multivariate survival analyses. Statistical significance was set at *P* < 0.05 for all tests.

## Results

### iRGD enhances NRP1-dependent entry of a coinjected tracer into colorectal cancers

**Figure 1.**
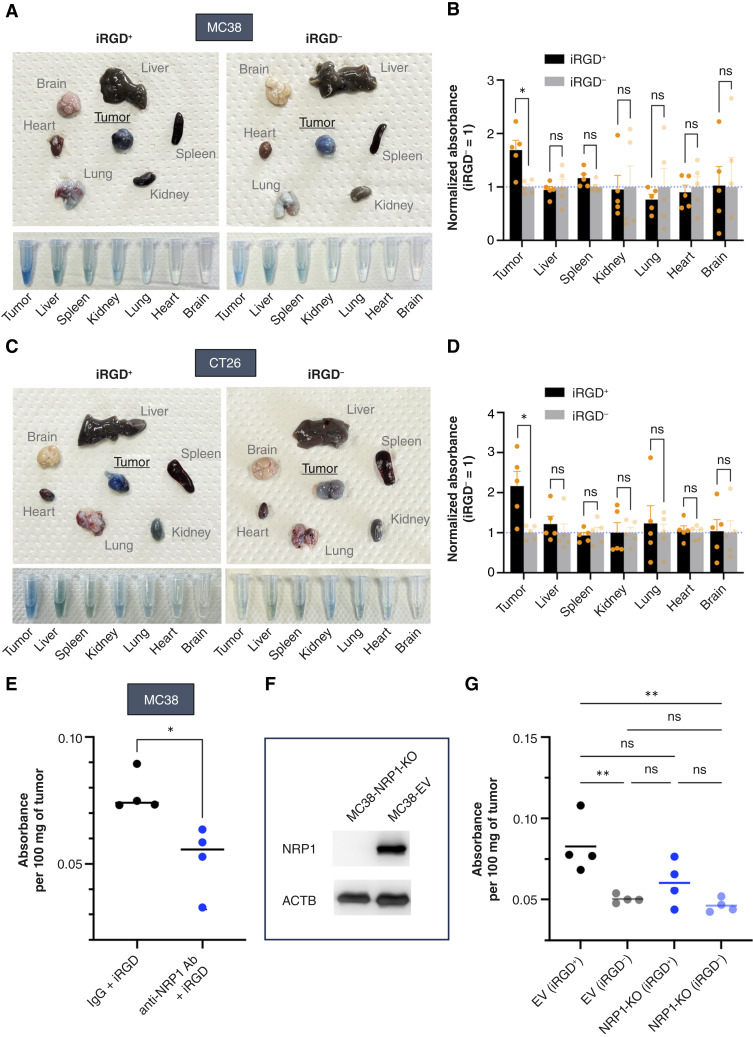
Assessment of iRGD-mediated drug accumulation in tumors using Evans blue dye as a tracer. Mice bearing MC38 or CT26 tumors were intravenously injected with 50 mg/kg of Evans blue, followed by 12.5 μmol/kg iRGD or PBS alone after 5 minutes. Tissues were collected after 30 minutes of circulation and subsequent PBS perfusion. **A** and **C,** Representative images of tumors and major organs after Evans blue administration with or without iRGD. **B** and **D,** Normalized absorbance values of iRGD^+^ and iRGD^−^ for tumor and each organ. Absorbance values were background-corrected using mice not injected with Evans blue and normalized to the mean value of the iRGD^−^ group. **E,** Dye accumulation in MC38 tumors treated with iRGD and either isotype control IgG or anti-NRP1 antibody (*n* = 4 per group). **F,** Western blot analysis confirming the loss of NRP1 expression in MC38-NRP1-KO cells compared with MC38-EV (EV control) cells. β-Actin (ACTB) was used as the loading control. **G,** Dye accumulation in MC38-EV and MC38-NRP1-KO tumors, with or without iRGD treatment (*n* = 4 per group). Data are presented as mean ± SEM. Statistical analyses were performed using multiple *t* tests, the Welch *t* test, or one-way ANOVA as appropriate. *, *P* < 0.05; **, *P *< 0.01; ns, not significant.

We performed systemic permeability assays to investigate whether iRGD treatment enhanced tumor-specific delivery of the coadministered agents to colorectal tumors. Evans blue dye was injected systemically, followed by iRGD administration in the indicated groups, into mice bearing subcutaneous MC38 tumors. Visual inspection revealed increased blue coloration of the tumors in iRGD-treated mice (Fig. 1A). Absorbance values corrected for background absorbance and normalized to tumor weight were higher in tumors than in other organs when iRGD was coadministered, showing significantly greater tumor-selective dye accumulation compared with controls (Fig. 1B). Similar results were obtained using a second colorectal cancer mouse model prepared using CT26 cells (Fig. 1C and D). iRGD enhanced dye entry into tumors in an NRP1-dependent manner because an anti-NRP1 antibody inhibited this effect (Fig. 1E). To further evaluate NRP1 dependency, we engineered MC38-NRP1-KO cells and confirmed the loss of NRP1 expression using immunoblot assays (Fig. 1F). In the absence of iRGD, both EV and NRP1-KO tumors showed low Evans blue uptake. In the EV group, similar to the parental cells, Evans blue uptake was significantly enhanced by the combined administration of iRGD. In the NRP1-KO group, although there was no significant difference, some tumors still showed increased Evans blue uptake with iRGD treatment (Fig. 1G).

### iRGD potentiates the antitumor effect of anti–PD-1 antibody treatment in murine colorectal cancer models

Anti–PD-1 antibodies are gaining attention as promising therapeutic agents for colorectal cancer, especially in MSI-H tumors (25–27). Based on the systemic permeability assay results, we evaluated whether iRGD coadministration enhanced the antitumor efficacy of the anti–PD-1 antibody in murine colorectal cancer models.

**Figure 2.**
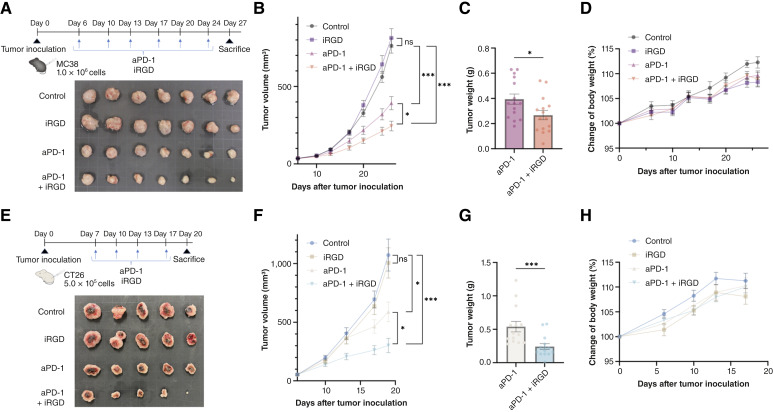
Tumor growth inhibition by anti–PD-1 antibody (aPD-1) and iRGD was evaluated using pooled data from independent experiments in MC38 (*n* = 15) and CT26 (*n* = 14) subcutaneous tumor models. Mice received intraperitoneal injections of PBS or aPD-1 (50 μg/mouse for MC38; 100 μg/mouse for CT26) and intravenous injections of iRGD (25 μmol/kg) twice weekly. **A** and **E,** Experimental timelines and representative tumors at the endpoint. **B** and **F,** Tumor growth curves. **C** and **G,** Tumor weight at the endpoint in mice treated with aPD-1 or aPD-1 plus iRGD. **D** and **H,** Change in body weight over the experimental period. Tumor volume was calculated as (length × width^2^)/2. Data are presented as mean ± SEM. Statistical analyses were performed using the Welch *t* test, the Mann–Whitney U test, or two-way ANOVA, as appropriate. *, *P* < 0.05; ***, *P* < 0.001; ns, not significant.

In mice bearing subcutaneous MSI-H MC38 tumors (28, 29), the greatest tumor reduction was observed in the combination group treated with iRGD and anti–PD-1 antibody (Fig. 2A). Pooled analyses demonstrated that tumor growth was significantly suppressed in the combination group compared with anti–PD-1 monotherapy (Fig. 2B) and tumor weight at sacrifice was also significantly reduced (Fig. 2C). Consistent with the results of previous studies, iRGD alone showed no detectable antitumor effects (3, 30). iRGD did not enhance off-target toxicities based on body weight measurements, consistent with its tumor-specific activities (Fig. 2D).

Similarly, in mice bearing subcutaneous MSS CT26 tumors (28, 31), a significant reduction in both tumor volume and weight was observed in the combination group compared with anti–PD-1 monotherapy (Fig. 2E–G). Body weight changes were comparable among treatment groups (Fig. 2H). These findings suggest that the addition of iRGD enhances the therapeutic activity of anti–PD-1 antibody against MSI-H colorectal cancer tumors and, potentially, MSS cancers.

### Increased abundance of and functional alterations in intratumoral CD8^+^ T cells by iRGD and anti–PD-1 combination therapy

The therapeutic efficacy of ICIs has been associated with the abundance and functional state of intratumoral immune cells, particularly CD8^+^ T cells, including their proliferative capacity and effector activity (32–34). Therefore, to assess the immunologic effects of iRGD in combination with anti–PD-1 therapy, we quantified intratumoral CD8^+^ T cells and evaluated markers of proliferation and cytotoxic activation.

**Figure 3.**
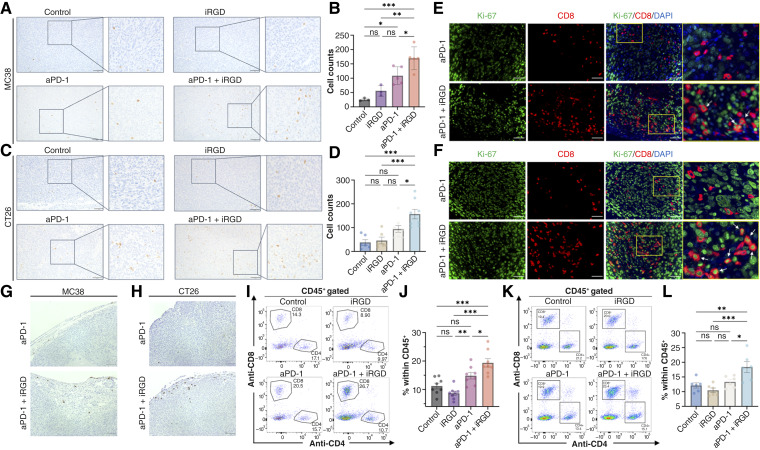
Tumor tissues from MC38- and CT26-bearing mice were analyzed by IHC, immunofluorescence, and flow cytometry to assess the abundance and proliferative status of intratumoral CD8^+^ T cells. **A** and **C,** Representative IHC images showing CD8^+^ cells in MC38 and CT26 tumor sections. **B** and **D,** Quantification of CD8^+^ cells per tumor, calculated as the average of 10 random fields per tumor. In MC38 (**B**), *n* = 3 for control and iRGD groups and *n* = 5 for anti–PD-1 antibody (aPD-1) and aPD-1 + iRGD groups; in CT26 (**D**), *n* = 6 for control and iRGD groups and *n* = 8 for aPD-1 and aPD-1 + iRGD groups. **E** and **F,** Representative immunofluorescence images showing Ki-67 (green) and CD8 (red) staining in MC38 (**E**) and CT26 (**F**) tumors treated with aPD-1 or aPD-1 plus iRGD. Nuclei were counterstained with DAPI (blue). Merged images and higher-magnification views of the boxed areas. Arrows indicate CD8^+^ cells exhibiting strong Ki-67 expression. **G** and **H,** Representative IHC images showing granzyme B–positive cells in MC38 (**G**) and CT26 (**H**) tumor sections treated with aPD-1 or aPD-1 plus iRGD. **I** and **K,** Representative flow cytometry plots showing CD4^+^ and CD8^+^ T-cell populations gated on CD45^+^ cells. **J** and **L,** Percentages of CD8^+^ T cells (MC38, *n* = 8; CT26, *n* = 6). Data are presented as mean ± SEM. Statistical analyses were performed using one-way ANOVA. *, *P* < 0.05; **, *P* < 0.01; ***, *P* < 0.001; ns, not significant. Scale bars, 100 μm (**A**, **C**, **G**, and **H**) and 50 μm (**E** and **F**).

In both MC38 and CT26 models, IHC revealed that CD8^+^ T cells were significantly increased in the iRGD/anti–PD-1 combination group compared with the other treatment groups (Fig. 3A–D). Immunofluorescent staining for CD8 and Ki-67 demonstrated that in both MC38 and CT26 tumors, the frequency of Ki-67–positive CD8^+^ T cells was increased in the iRGD/anti–PD-1 combination group compared with anti–PD-1 monotherapy (Fig. 3E and F). In addition, granzyme B–positive cells were mainly observed at the tumor periphery and were increased in the combination group compared with anti–PD-1 monotherapy (Fig. 3G and H).

Flow cytometry analysis further supported these findings. In both MC38 and CT26 models, the proportion of CD8^+^ T cells within the CD45^+^ immune cell population was significantly higher in the combination group compared with all other treatment groups, including anti–PD-1 monotherapy (Fig. 3I–L), consistent with the enhanced antitumor effects observed in the treatment studies. The proportion of CD45^+^ cells tended to be highest in the combination group in the MC38 model (Supplementary Fig. S1A and S1B), whereas no significant differences were observed among treatment groups in the CT26 model (Supplementary Fig. S1C and S1D). The proportion of regulatory T cells (CD4^+^CD25^+^Foxp3^+^) did not differ significantly among treatment groups in either model (Supplementary Fig. S1E–S1H). Taken together, these results indicate that the addition of iRGD to anti–PD-1 therapy is associated with increased abundance and functional activation of intratumoral CD8^+^ T cells.

### Deleting NRP1 expression in colorectal cancer cells does not abolish the effects of iRGD/anti–PD-1 combination therapy

**Figure 4.**
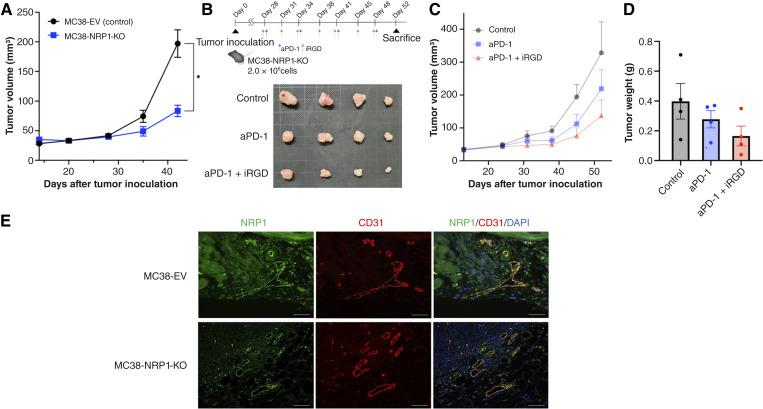
Tumor growth of MC38 tumors with or without NRP1 deletion and response of NRP1-KO tumors to iRGD plus anti–PD-1 (aPD-1) therapy. **A,** Tumor growth curves of MC38-EV (control) and MC38-NRP1-KO tumors (*n* = 5 per group). **B–D,** Therapeutic efficacy of iRGD in mice bearing MC38-NRP1-KO tumors. **B,** Treatment schedule and representative images of excised tumors from mice treated with PBS (control), aPD-1, or aPD-1 plus iRGD. **C,** Tumor growth curves and (**D**) tumor weights at endpoint (*n* = 4 per group). **E,** Representative immunofluorescence images of MC38-EV and MC38-NRP1-KO tumors stained for NRP1 (green), CD31 (red), and DAPI (blue). NRP1 expression is markedly reduced in tumor cells of NRP1-KO tumors but remains detectable in CD31^+^ vascular endothelial cells. Statistical analyses were performed using one-way or two-way ANOVA, as appropriate. *, *P* < 0.05. Scale bars, 50 μm.

Next, we investigated the role of NRP1 in the antitumor effects of the combination therapy. We hypothesized that the enhanced efficacy was driven by the NRP1-dependent penetration of the anti–PD-1 antibody induced by iRGD. Thus, we examined the efficacy of the iRGD/anti–PD-1 combination therapy in mice with subcutaneous MC38-NRP1-KO tumors. Notably, MC38-NRP1-KO tumors grew significantly slower than MC38-EV control tumors, suggesting a role for NRP1 in colorectal cancer progression (Fig. 4A). Contrary to our expectation that iRGD effects would be abolished in NRP1-KO tumors, there remained a tendency for tumors in the combination arm to have reduced volume and weight (Fig. 4B–D). Immunofluorescence analysis of MC38-NRP1-KO tumors revealed preserved NRP1 expression in CD31^+^ blood vessels and, to some extent, in surrounding stromal cells, which presumably contributed to the residual effect of iRGD (Fig. 4E).

### NRP1 is widely expressed in human colorectal cancer

**Figure 5.**
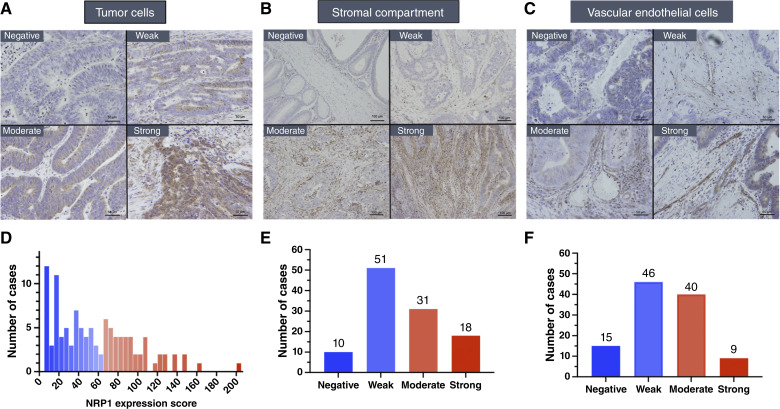
NRP1 expression in tumor cells, the stromal compartment, and vascular endothelial cells was assessed by IHC in colorectal cancer tissues. Representative images of NRP1 IHC staining in tumor cells (**A**), the stromal compartment (**B**), and vascular endothelial cells (**C**), showing 4 levels of staining intensity (negative, weak, moderate, and strong). Distribution of NRP1 expression scores in tumor cells (**D**) and the frequencies of each staining category in the stromal compartment (**E**) and vascular endothelial cells (**F**). Scale bars, 50 μm (**A** and **C**) and 100 μm (**B**).

To assess the clinical applicability of iRGD, we examined NRP1 expression in human colorectal cancer tissues. IHC analysis of samples from 110 patients with colorectal cancer revealed varying degrees of NRP1 expression in tumor epithelial and stromal compartments (Fig. 5A and B). To further characterize the stromal compartment, vascular structures were separately evaluated (Fig. 5C). Patients were classified into high- and low-expression groups based on the criteria for each compartment. NRP1 expression in tumor cells was detected across all cases, with heterogeneous distribution (Fig. 5D). In the stromal compartment, NRP1 expression was detected in more than 90% of cases, and approximately 45% of cases showed moderate to strong staining (Fig. 5E). In vascular endothelial cells, a similar distribution pattern to that in the stromal compartment was observed (Fig. 5F). Serial sections with CD31 and α-SMA staining were examined in representative cases (Supplementary Fig. S2). NRP1 expression was observed in both vascular-associated and nonvascular stromal areas, with staining intensity tending to be higher in vascular-associated areas compared with nonvascular stromal (CAF-rich) areas.

### Stromal expression of NRP1 correlates with poor prognosis in colorectal cancer

**Figure 6.**
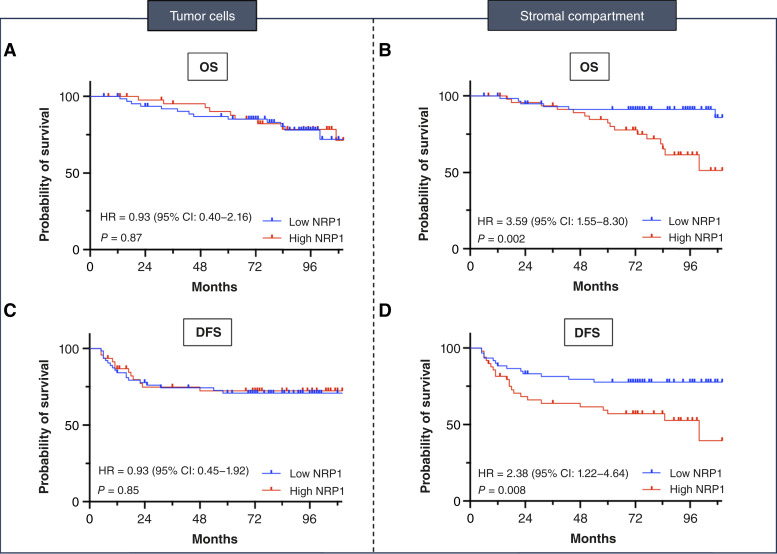
Kaplan–Meier survival curves for OS and DFS based on NRP1 expression scores in tumor cells (**A** and **C**) and the stromal compartment (**B** and **D**). Although no significant differences are observed for tumor cell NRP1 expression, high stromal NRP1 expression is significantly associated with shorter OS (*P* = 0.002) and DFS (*P* = 0.008). *P* values were calculated using the log-rank test.

Kaplan–Meier survival analysis showed that high NRP1 expression in the stromal compartment, rather than in tumor cells, was significantly associated with shorter overall survival (OS; tumor: Fig. 6A; *P* = 0.87, stromal: Fig. 6B; *P* = 0.002) and disease-free survival (DFS; tumor: Fig. 6C; *P* = 0.85, stromal: Fig. 6D; *P* = 0.008). Tumor cell NRP1 expression showed no significant association with tumor-specific survival (Supplementary Fig. S3A; *P* = 0.34), whereas stromal NRP1 expression was associated with poor outcomes (Supplementary Fig. S3B; *P* = 0.015). NRP1 expression in vascular endothelial cells was significantly associated with shorter OS (Supplementary Fig. S3C; *P* = 0.012) and showed a trend toward shorter DFS (Supplementary Fig. S3D; *P* = 0.094). According to clinicopathologic characteristics stratified by stromal NRP1 expression, well-differentiated tumors more frequently exhibited high stromal NRP1 expression (*P* = 0.013), whereas female patients were more likely to exhibit low stromal NRP1 expression (*P* = 0.002; Supplementary Table S1). Variables that were significant in the univariate analysis (*P* < 0.05) were entered into a multivariate Cox regression analysis. In this model, both nonadjuvant chemotherapy [hazard ratio (HR) = 2.634; 95% confidence interval (CI), 1.065–6.513; *P* = 0.036] and high stromal NRP1 expression (HR = 2.783; 95% CI, 1.040–7.452; *P* = 0.042) were independently associated with poor OS (Table 1).

**Table 1.** Cox proportional hazards model of prognostic factors in patients with colorectal cancer.

Factor	Univariate analysis			Multivariate analysis		
	HR	95% CI	*P* value	HR	95% CI	*P* value
Age (years; ≥60 vs. <60)	1.209	0.474–3.083	0.691	NA	NA	NA
Gender (female vs. male)	1.641	0.673–3.998	0.276	NA	NA	NA
Tumor size (mm; ≥50 vs. <50)	1.554	0.681–3.543	0.295	NA	NA	NA
Histologic type (por/muc vs. tub)	0.882	0.206–3.769	0.865	NA	NA	NA
T stage (T3/T4 vs. T1/T2)	1.535	0.360–6.553	0.563	NA	NA	NA
N stage (N2 vs. N1)	3.103	1.338–7.197	0.008	2.049	0.853–4.920	0.109
Lymphatic invasion (ly+ vs. ly−)	1.698	0.697–4.138	0.244	NA	NA	NA
Vascular invasion (v+ vs. v−)	1.060	0.416–2.702	0.903	NA	NA	NA
Adjuvant chemotherapy (no vs. yes)	4.157	1.790–9.656	<0.001	2.634	1.065–6.513	0.036
Tumor NRP1 expression (high vs. low)	0.931	0.401–2.166	0.869	NA	NA	NA
Stromal NRP1 expression (high vs. low)	4.027	1.556–10.422	0.004	2.783	1.040–7.452	0.042

Abbreviations: muc, mucinous adenocarcinoma; N, node; NA, not applicable; por, poorly differentiated adenocarcinoma; T, tumor; tub, tubular adenocarcinoma.

## Discussion

In this study, we demonstrated that iRGD enhances intratumoral delivery of a coinjected agent into colorectal cancer tumors in an NRP1-dependent manner. We further showed that combination therapy with iRGD and anti–PD-1 antibody effectively suppressed tumor growth in murine colorectal cancer models and was associated with an increased abundance of intratumoral CD8^+^ T cells exhibiting enhanced proliferative and effector-associated features. Deletion of NRP1 expression in colorectal cancer cells was not sufficient to abolish the effects of iRGD on intratumoral dye accumulation or therapeutic response to anti–PD-1 therapy, suggesting that stromal NRP1 may play a critical role in mediating iRGD function. Analysis of clinical colorectal cancer specimens revealed widespread NRP1 expression in both tumor and stromal compartments. Notably, stromal NRP1 expression, including tumor-associated blood vessels, was associated with poor prognosis.

iRGD selectively enhanced the tumor entry of coadministered Evans blue dye without affecting normal tissues, which is in agreement with the tumor-specific activity of iRGD. This effect was NRP1 dependent because a blocking antibody significantly reduced iRGD-mediated dye uptake. Importantly, genetic ablation of NRP1 expression in tumor cells did not completely inhibit intratumoral delivery, suggesting the involvement of stromal NRP1 in this effect. These findings are in line with the known expression profile of NRP1 in angiogenic vasculature and its role in mediating vascular permeability (35–38).

**Figure 7.**
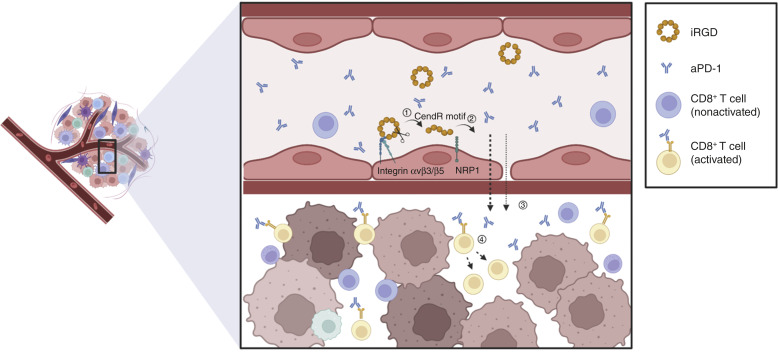
Proposed mechanism of iRGD-enhanced anti–PD-1 therapy. (i) CendR motif exposure and NRP1 binding. (ii) NRP1-dependent increase in vascular permeability. (iii) Enhanced intratumoral delivery of anti–PD-1 (aPD-1) antibody. (iv) Activation and proliferation of intratumoral CD8^+^ T cells. [Created in BioRender. Maeda, M. (2026) https://BioRender.com/bwlwni4.]

The finding that the iRGD/anti–PD-1 antibody combination therapy significantly reduced tumor growth in both MSI-H and MSS colorectal cancer mouse models has important implications. The clinical success of ICIs in colorectal cancer has been limited to MSI-H tumors, which represent only a small proportion of patients. For the much larger population with MSS colorectal cancer, therapeutic options remain limited (25). Our data suggest that iRGD may facilitate responsiveness under certain conditions of MSS colorectal cancer to ICIs by breaking through the physical and stromal barriers that underlie immune exclusion (3, 39), enabling ICIs to reach and activate effector T cells within the tumor microenvironment. Indeed, iRGD/anti–PD-1 antibody combination therapy significantly increased the number of intratumoral CD8^+^ T cells and enhanced their functional state in both the MSI-H and MSS models. These findings suggest that NRP1-mediated vascular permeability enhances intratumoral delivery of anti–PD-1 antibody, thereby promoting CD8^+^ T-cell activation and proliferation within the tumor microenvironment (Fig. 7). A recent study in hepatocellular carcinoma models similarly reported enhanced intratumoral CD8^+^ T-cell activation following iRGD coadministration with anti–PD-L1 therapy (40). However, although the MC38 and CT26 models used in this study are widely employed, they do not fully recapitulate the molecular and immunologic characteristics of human MSI-H and MSS colorectal cancers, respectively, especially when established as subcutaneous tumors (41, 42). Thus, further validation in orthotopic or genetically engineered models that more precisely represent the phenotype of human MSI-H and MSS diseases is warranted.

Consistent with the Evans blue study, tumor cell–specific deletion of NRP1 did not eliminate the antitumor effect of iRGD/anti–PD-1 antibody combination therapy in colorectal tumor–bearing mice. Residual NRP1 expression persisted in the vascular and stromal compartments of tumors, which likely contributed to the residual effects of iRGD. Thus, NRP1 expression in the stroma, in addition to the epithelial compartment, may be critical in mediating iRGD effects. However, the present findings are based on indirect evidence, as NRP1 deletion was limited to tumor cells. Definitive evaluation of stromal NRP1 would require cell type–specific KO models targeting individual stromal compartments. Although technically challenging, this represents an important direction for future investigation.

IHC analysis of human colorectal cancer specimens revealed widespread expression of NRP1 in both epithelial and stromal compartments, including the tumor vasculature, suggesting that iRGD-based therapy may have broad applicability in colorectal cancer. In our patient cohort, strong stromal NRP1 expression, including that in the tumor vasculature, correlated with shorter survival, supporting stromal NRP1 as a negative prognostic factor. These findings suggest that iRGD-based therapies may offer a potential therapeutic strategy in colorectal cancer. Our conclusions are based on the analysis of a relatively homogeneous cohort and compartment-specific profiling of NRP1 expression in tumor tissues. In contrast, previous reviews and meta-analyses have not demonstrated a consistent association between NRP1 expression and colorectal cancer prognosis (20). This inconsistency may be attributable to methodologic variability, lack of compartment-specific evaluation, and heterogeneity in patient cohorts. Previous studies employed diverse approaches, including IHC, transcriptomic analyses using public datasets (43), and measurement of serum NRP1 (44, 45), rather than direct compartment-specific assessment in tumor tissue. Moreover, many reported cohorts included patients with distant metastases, making it difficult to exclude the influence of confounding factors beyond NRP1 expression.

The finding that NRP1 expression in tumor cells was not associated with prognosis was surprising because tumor cell–specific NRP1 deletion in our MC38 mouse model led to significantly slower tumor growth compared with controls. NRP1 on tumor cells is indeed known to facilitate cancer progression through pleiotropic effects on the tumor microenvironment, such as paracrine activation of endothelial vascular endothelial growth factor receptor 2 signaling (46, 47) and direct regulation of tumor cell functions such as cell adhesion and migration (48, 49). The limited stromal burden in subcutaneous murine models (50, 51) and selective deletion of NRP1 expression in the tumor cells likely revealed the contribution of tumor cell NRP1 in our animal study. In contrast, in human colorectal cancers, the role of tumor cell NRP1 may be masked given the relatively rich stroma. In our patient cohort, we frequently observed cases with low tumor cell NRP1 expression but high NRP1 expression in the densely infiltrating stromal tissue. Thus, the prognostic impact of tumor cell NRP1 expression may be overshadowed by the dominant contribution of stromal NRP1 in human colorectal cancer. Further clinical validation in larger, prospective datasets and additional animal studies using clinically relevant orthotopic colorectal cancer mouse models are warranted.

### Conclusions

Our study showed that combining the tumor-penetrating peptide iRGD and anti–PD-1 antibody therapy provided significant therapeutic effects in colorectal cancer models, likely mediated by stromal NRP1, particularly in the tumor vasculature. Stromal NRP1 expression had high clinical value, as it predicted poor prognosis in patients with colorectal cancer. This finding supports its relevance as a therapeutic target for enhancing intratumoral drug delivery and provides a rationale for further investigation of iRGD-based combination strategies in colorectal cancer.

## Supplementary Material

Supplementary Table S1 Correlation between stromal NRP1 expression and clinicopathological characteristics of CRC patients.

Supplementary Figure S1 Flow cytometry analysis of CD45^+^ cells and regulatory T cells.

Supplementary Figure S2 NRP1 expression patterns within stromal regions in human colorectal cancer tissues.

Supplementary Figure S3 Survival analysis according to NRP1 expression across tumor compartments.

## Data Availability

The data generated in this study are available upon request to the corresponding author.
