# Supplementary material for: Enhancing Immunotherapeutic Response in Colorectal Cancer with a Neuropilin 1–Targeting Tumor-Penetrating Peptide
Source: Cancer Res Commun. 2026 Jun 10;6(6):1364–75. doi: 10.1158/2767-9764.CRC-25-0619 (PMC13250810; doi:10.1158/2767-9764.CRC-25-0619)
Supplement: Supplementary Table S1 — Correlation between stromal NRP1 expression and clinicopathological characteristics of CRC patients. [file crc-25-0619_supplementary_table_s1_suppst1.docx]

Total patients

Age at surgery (years)

< 60

≧ 60

Gender

Male

Female

Performance status

0-1

2-3

Preoperative treatment

Yes

No

Tumor size

< 50

≧ 50

Histological type

tub

por, muc

T stage

T1, T2

T3, T4

N stage

N1

N2

Lymphatic invasion

Positive

Negative

Vascular invasion

Positive

Negative

Adjuvant chemotherapy

Yes

No

**Supplementary Table S1.** Correlation between stromal NRP1 expression and clinicopathological characteristics of CRC patients.

110

27

83

65

45

106

4

10

100

57

53

99

11

13

97

84

26

23

87

78

32

69

41

49

12

37

37

12

46

3

2

47

25

24

48

1

9

40

35

14

9

40

34

15

26

23

61

15

46

28

33

60

1

8

53

32

29

51

10

4

57

49

12

14

47

44

17

43

18

**Characteristics**

**All cases**

**High**

**Low**

<0.001

9.854

1.559

2.683

0.023

6.219

3.637

1.192

0.345

0.099

3.531

**χ2**

***P-value***

0.990

0.002

0.212

0.101

0.881

0.013

0.057

0.275

0.557

0.753

0.060

**NRP1 level**
