# Supplementary material for: Enhancing Immunotherapeutic Response in Colorectal Cancer with a Neuropilin 1–Targeting Tumor-Penetrating Peptide
Source: Cancer Res Commun. 2026 Jun 10;6(6):1364–75. doi: 10.1158/2767-9764.CRC-25-0619 (PMC13250810; doi:10.1158/2767-9764.CRC-25-0619)
Supplement: Supplementary Figure S1 — Flow cytometry analysis of CD45+ cells and regulatory T cells. [file crc-25-0619_supplementary_figure_s1_suppsf1.docx]

**Supplementary Figure S1 Flow cytometry analysis of CD45⁺ cells and regulatory T cells.**

MC38 tumors **(A, B, E, F)** and CT26 tumors (**C**, **D**, **G**, **H**) were analyzed.

**(A, C)** Representative plots and **(B, D)** corresponding bar graphs showing the percentage of CD45⁺ cells among live cells (MC38, n = 8; CT26, n = 6). **(E, G)** Representative plots and **(F, H)** corresponding bar graphs showing the frequency of Tregs (CD4⁺CD25⁺FOXP3⁺) within CD4⁺ cells (MC38, n = 6; CT26, n = 3). Data are presented as mean ± SEM. Statistical analyses were performed using one-way ANOVA. **P* < 0.05, ****P* < 0.001; ns, not significant.
