# Supplementary material for: Enhancing Immunotherapeutic Response in Colorectal Cancer with a Neuropilin 1–Targeting Tumor-Penetrating Peptide
Source: Cancer Res Commun. 2026 Jun 10;6(6):1364–75. doi: 10.1158/2767-9764.CRC-25-0619 (PMC13250810; doi:10.1158/2767-9764.CRC-25-0619)
Supplement: Supplementary Figure S2 — NRP1 expression patterns within stromal regions in human colorectal cancer tissues. [file crc-25-0619_supplementary_figure_s2_suppsf2.docx]

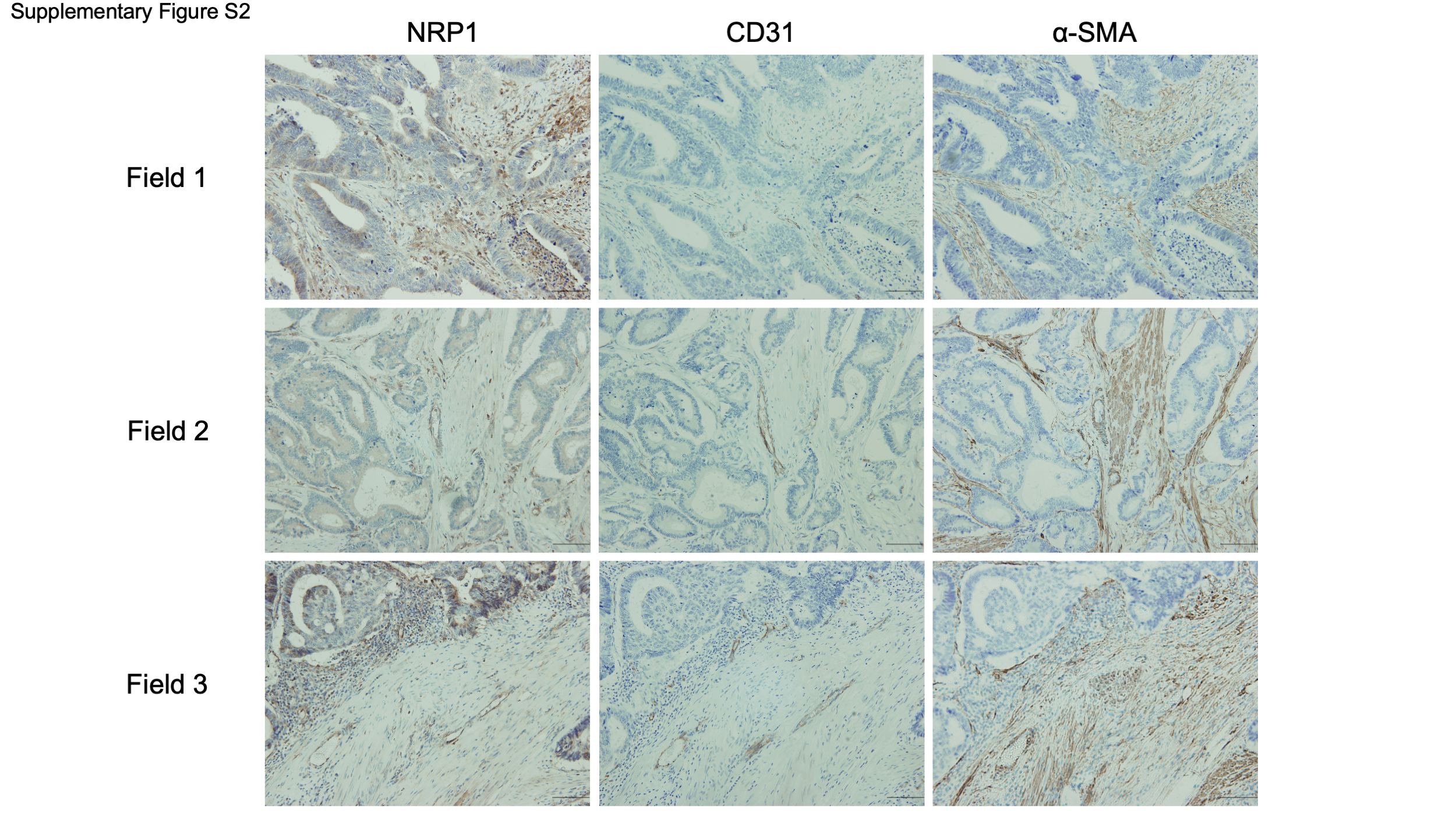


**Supplementary Figure S2 NRP1 expression patterns within stromal regions in human colorectal cancer tissues.**

Serial sections showing NRP1 expression in relation to vascular and stromal components in human colorectal cancer tissues. Sections were stained for NRP1, CD31, and α-SMA. Three fields from independent cases are shown, with each row representing corresponding areas from the same case. NRP1 expression is observed in both vascular-associated and nonvascular stromal areas (CAF-rich), with relatively stronger staining in vascular-associated areas. Scale bar, 100 μm.
