# Supplementary material for: Enhancing Immunotherapeutic Response in Colorectal Cancer with a Neuropilin 1–Targeting Tumor-Penetrating Peptide
Source: Cancer Res Commun. 2026 Jun 10;6(6):1364–75. doi: 10.1158/2767-9764.CRC-25-0619 (PMC13250810; doi:10.1158/2767-9764.CRC-25-0619)
Supplement: Supplementary Figure S3 — Survival analysis according to NRP1 expression across tumor compartments. [file crc-25-0619_supplementary_figure_s3_suppsf3.docx]

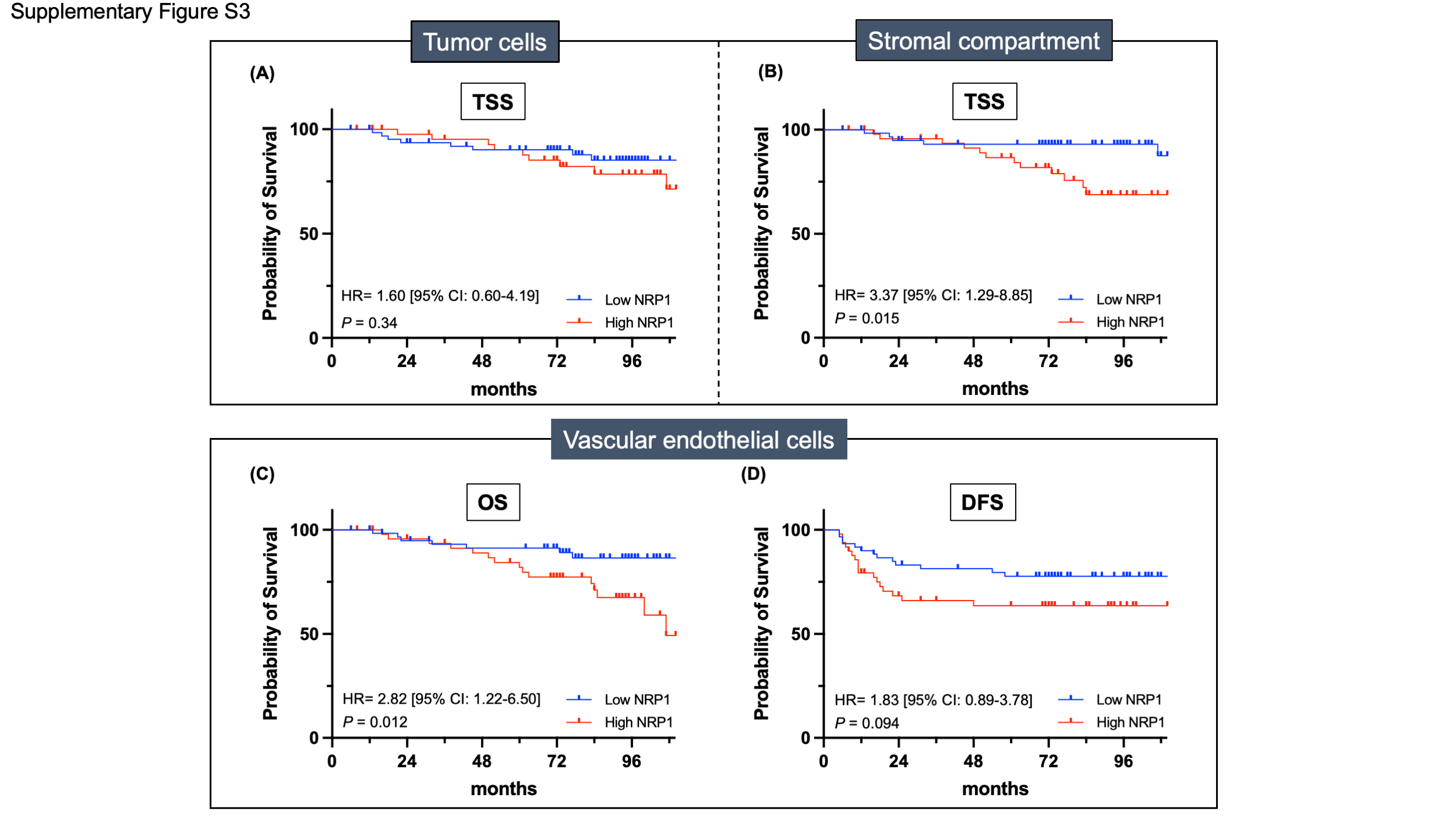


**Supplementary Figure S3 Survival analysis according to NRP1 expression across tumor compartments.**

Kaplan–Meier curves of tumor-specific survival (TSS) according to NRP1 expression in tumor cells (A) and stromal compartments (B). High stromal NRP1 expression was significantly associated with shorter TSS (P = 0.015), while high tumor cell NRP1 expression showed no significant association with TSS (P = 0.34). Kaplan–Meier curves based on NRP1 expression in vascular endothelial cells are shown for overall survival (OS) (C) and disease-free survival (DFS) (D). High endothelial NRP1 expression was associated with shorter OS (P = 0.012) and showed a trend toward shorter DFS (P = 0.094). P-values were calculated using the log-rank test.
